# Caffeine Supplementation Increases Muscle Strength, but Not Endurance, While Both Caffeine and Its Expectation Elevate Blood Lactate: A Balanced-Placebo Design Study

**DOI:** 10.3390/nu18050801

**Published:** 2026-02-28

**Authors:** Edgard Melo Keene Von Koenig Soares, Carlos Janssen Gomes da Cruz, Stephen J. Ives, Guilherme Eckhardt Molina, Keila Elizabeth Fontana

**Affiliations:** 1 Department of Health and Human Physiological Sciences, Skidmore College, Saratoga Springs, NY 12866, USA; 2 Exercise Physiology Laboratory, Universidade de Brasília, Brasilia 70910-900, DF, Brazil; 3 Cardiac Autonomic Function Research Group, Centro Universitário Euro Americano-UNIEURO, Brasilia 71900-100, DF, Brazil

**Keywords:** resistance exercise, isokinetic dynamometry, torque production, placebo effect, anaerobic metabolism

## Abstract

**Objectives**: To determine whether caffeine supplementation and its expectancy influence muscle strength (ST) and muscle endurance (ME) using a balanced-placebo design. **Methods:** Using a randomized, double-blind, balanced-placebo design, resistance-trained men (18–30 years; n = 16) participated in two assessment/familiarization visits (demographics; one repetition maximum (1RM) followed by four counterbalanced sessions: C/C (informed caffeine/used caffeine), P/C (informed placebo/used caffeine), C/P (informed caffeine/used placebo), P/P (informed placebo/used placebo). Caffeine dose was 5 mg/kg. Peak torque (PT) and maximum work (MW) were measured in the knee extensors at 0, 60, 180, and 300°/s, which was followed by ME testing (three sets of maximal repetitions using 60%1RM). Capillary blood lactate was measured after ME testing. **Results:** Caffeine increased PT only during static and 60°/s contractions (4%; *p* ≤ 0.003; d = 0.3 for both speeds), while MW increased across all speeds (4%; *p* < 0.001; d = 0.2). Caffeine did not increase ME (3%; *p* = 0.184; d = 0.1), but it did increase blood lactate levels (1.2 mmol/L; *p* < 0.001; d = 0.7). Caffeine expectation did not improve ST or ME, but increased blood lactate levels (0.7 mmol/L; *p* = 0.003; d = 0.4). Across ST and ME, responses to caffeine were markedly heterogeneous, with 50–88% of individuals considered responders (∆ > 0), and improvements in responders ranged from 1–16%. **Conclusions:** Caffeine significantly increased ST, despite ME increasing in 50% of participants, this was not significant. No placebo effect was observed in ST or ME, but it significantly increased lactate. Further research is necessary to elucidate the mechanisms behind this variability in the ME response, especially the role of fiber-type predominance.

## 1. Introduction

Caffeine (1,3,7-trimethylxanthine) is the world’s most widely consumed psychoactive substance, and is widely recognized for its ergogenic benefits in physical performance [1,2,3,4,5], with 46–74% of athletes reporting its use during international and national competitions [6,7]. An interesting example can be observed in elite English soccer clubs, where a survey indicated that 97% of clubs supply caffeine to players to enhance their performance [8]. The benefit of caffeine is not limited to professional athletes, with abundant data across various cohorts [4,9,10].

Caffeine is best known for enhancing endurance-related exercise performance [11]. However, numerous studies also show its capacity to increase maximum strength and muscular endurance acutely [10,12], with a meta-analysis reporting small effect sizes (Cohen’s d) in both maximum strength and muscle endurance, 0.2 and 0.3, respectively [3]. In contrast to the apparent consistency across meta-analyses, several caffeine supplementation studies consistently report heterogeneous results: some have found positive effects [4,9,13,14,15,16,17], while others have not [18,19,20,21]. The aforementioned studies exhibit similarities in caffeine dosage and supplementation timing, and, in some instances, exercise protocols, thereby leaving an open gap for further investigation into the reasons why improvements are observed in some cases while not in others. This heterogeneity of findings may be explained by the sensitivity of the instrument or task used to measure performance, the training level of the volunteers evaluated, the muscle group used, the type of muscle contraction, the type of test used, age, sex, individual capacity to metabolize caffeine (e.g., variations in *CYP1A2* gene), and to genetic variations of the adenosine A_2A_ receptor gene (*ADORA2A*) [1,3,11,12,22,23,24].

Amidst these controversies, another important factor emerges—the placebo effect. The placebo effect has been widely studied in the medical field related to analgesia [25]. It can be defined as a positive response, such as a decrease in pain, depression, and/or improvement in physical performance, when an inert substance, intervention, or treatment is applied [25,26]. Therefore, considering that one of the potential ergogenic mechanisms of caffeine involves the perception of reduced pain and effort [27], it is reasonable to hypothesize that positive expectations concerning caffeine may also induce a placebo effect, as seen in studies investigating the effect of caffeine expectation on multiple repetition protocols. [28,29,30]. A meta-analysis assessing the placebo effect on exercise performance [26] concluded that the placebo effect may be a significant factor in sports performance. In some real-world scenarios, small improvements may have great practical importance [26,31,32]; a small-to-moderate effect size induced by placebo (0.4)—as reported by the meta-analysis [26]—could be greater than caffeine’s previously reported small effect (0.2–0.3) on muscle strength and endurance [3]. A question then arises: May the increase in maximum strength or muscular endurance due to caffeine consumption be partially influenced by its expectancy (i.e., a placebo effect)?

The double-blind research model is typically used to control the placebo effect and, in theory, would inhibit performance improvement solely based on participant expectation. However, it still has shortcomings [25], with most research on the subject stemming from the medical field, specifically on analgesia [33] and, more recently, on physical performance [30]. A particular concern with the double-blind model and caffeine supplementation revolves around blinding. Due to its well-known effects and, sometimes, participant familiarity with using caffeine, double-blind research participants often accurately identify the caffeine trial. Depending on the dose, 31–100% participants have been able to accurately identify the caffeine trial in blinded studies [34,35,36]. Thus, a major concern, and recent research seems to support this notion, is that participants often modulate their behavior when they believe they did not receive an ergogenic aid and underperform in placebo trials [36,37]. Thus, it is possible that the effect of caffeine could be inflated by this underperformance in the placebo trial.

As additional evidence supporting careful control of expectancy effects, a recent sodium bicarbonate study [38] (a supplement often associated with improved exercise performance [39]) used a placebo/nocebo design to test whether expectations modulate physical performance. Briefly, participants ingested sodium bicarbonate across conditions but received different instructions indicating that the supplement could be inert, ergogenic, or deleterious. Although sodium bicarbonate induced alkalosis in all trials, performance improvements occurred only when participants were told the supplement would have a positive effect [38].

Considering that the overwhelming majority of studies used the double-blind design [3], the use of a different experimental design to measure the effect of caffeine is justified [31,32,37], aiming to distinguish whether the increase in performance is exclusively from its physiological effect or if it results from the expectation of performance-enhancing effects, the placebo effect [26,28]. A clear example of this is seen in a study that employed a balanced placebo design to assess the effect of caffeine on 1000-m running performance in middle-distance athletes and reported that believing to have ingested caffeine improved performance in the same manner as actually receiving caffeine [40]. Further studies that take into account the potential placebo effect when assessing the ergogenic effects of caffeine on muscle strength and muscle endurance are warranted [37,41].

If a substantial placebo effect is present under caffeine supplementation, it implies that the modest influence of caffeine on muscle strength and endurance may be minimal or negligible. Consequently, prior research findings obtained through double-blind methodologies may need reevaluation. Thus, we aimed to assess the effect of caffeine supplementation (5 mg/kg of body mass) on the maximal strength and muscular endurance of trained young men using the experimental design known as the “balanced placebo design” [31,32]. As a secondary aim, we also explored individual variability in response to caffeine supplementation and the placebo effect. We hypothesized that caffeine supplementation would significantly increase muscle strength and muscle endurance. Similarly, we hypothesized that the placebo effect would significantly improve physical performance, i.e., that caffeine expectation would increase muscle strength and muscle endurance. Lastly, we hypothesized that the effect of caffeine and its expectation would be heterogeneous among participants in both muscle strength and endurance.

## 2. Materials and Methods

### 2.1. Participants and Study Overview

A non-random sampling method was employed. A poster was placed on the University grounds with contact information to participate in the research. A priori sample size calculation using G*Power 3.1 determined a sample size of 12 participants to detect an effect size of 0.2 (α = 0.05; power of 0.80; four measurements; 0.85 of correlation among measures) for a repeated measures ANOVA (within factors) analysis of the primary aim. Our study aimed to recruit a larger sample to account for a possible heterogeneity in caffeine response. A total of 25 physically active young men who met all the inclusion criteria were recruited. Participants were between 18–30 years old, reportedly healthy without cardiometabolic conditions, non-smokers, not using any medication, engaged in resistance training for at least six months, familiar with the squat technique, lacking any type of injury that prevented them from exercising, without any known allergy or sensitivity to caffeine, not using anabolic steroids or performance enhancing supplements during the study, and abstaining from caffeine supplements for at least a week before testing. We recruited only resistance-trained men to enhance the reliability of the one-repetition maximum and muscle endurance tests, and also to minimize the variability of the caffeine response related to training status [42,43] and sex differences [44].

All participants provided written informed consent. Nine volunteers did not complete all testing days, resulting in a final sample of 16. Eight volunteers who withdrew reported a lack of time as the reason for not completing all testing sessions, as sessions had to be completed in the morning. One volunteer reported that his back was sore after performing the muscle endurance test for the first time and chose not to continue with the study. This research was approved by the Human Research Ethics Committee of the School of Health Sciences (CEP-FS) under CAAE: 44527715.0.0000.0030 and was done in accordance with the most recent revisions to the Declaration of Helsinki, with all recruited participants providing written informed consent. This study was retrospectively registered in the Brazilian Registry of Clinical Trials (ReBEC), Brazil (registration number: RBR-8h8hbtx). The ReBEC is recognized by the World Health Organization’s International Clinical Trials Registry Platform (ICTRP). This trial was not registered prospectively because, at the time the study was planned, it did not meet the local definition of a clinical trial as it did not involve any “clinical, pharmacological, or pharmacodynamic effects” [45]. Our study also did not align with mandatory trial registration prescribed by Brazilian regulations [46].

### 2.2. Experimental Design

The study consisted of six sessions, with a minimum of 2 days and a maximum of 2 weeks between experimental sessions to accommodate participants’ individual schedules while allowing sufficient rest. To ensure consistent recovery and preparedness for this research, participants were instructed to refrain from performing lower-body resistance training throughout the entire experiment and from engaging in strenuous upper-body resistance training, aerobic exercise, or playing sports 48 h prior to an experimental session. The first two sessions aimed to increase internal validity, repeatability, and obtain basic study data (e.g., anthropometrics), familiarizing the participant with the procedures and questionnaires (first session), and one control session had the same procedures as the experimental session, but without any supplementation being given. These two sessions were followed by four randomized experimental sessions. The experimental sessions were scheduled with an average of 5 days between sessions.

**Figure 1:**
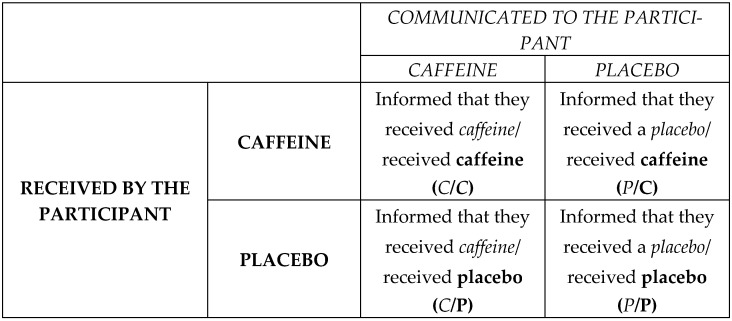
Schematic diagram of the placebo-balanced design. The first letter of each acronym represents the information given to the research participant, and the second letter is what the participant actually received. To facilitate understanding, the actual substance used is in bold, and the substance communicated to the participant is italicized.

This study employed a double-blind “balanced-placebo design” research model [31,32]. In all four experimental sessions, the participants received pills (containing a placebo or caffeine) without being able to discriminate between them. The researcher informed the participants that they were receiving caffeine in two different sessions and a placebo in the other two, while being blind to the real content of the pills. Thus, participants could receive caffeine and be informed that they were receiving caffeine in one session (C/C session) or receive caffeine while being told they were receiving a placebo (P/C session). Participants could also receive a placebo pill and be told that it was caffeine (C/P session) or receive a placebo while being told they were receiving a placebo (P/P session). Figure 1 shows a diagram to facilitate the understanding of the experimental sessions.

The order of the experimental sessions was randomized. One of the researchers (K.E.F.) was responsible for randomizing the order of the experimental sessions. Four session orders were prespecified, with the exception that caffeine sessions were separated by one placebo session to maximize washout (e.g., C/P, P/C, P/P, and C/C). Each order was assigned a unique number using a random number generator (1–4), and participants were allocated to orders sequentially based on enrollment order. The same researcher (K.E.F.) would calculate the correct supplement dosage and request that a lab technician assemble four packages (one for each experimental session), each labeled with the letter corresponding to the order of the sessions (A for the first, B for the second, and so on), which were confirmed before data collection by the lead researcher (E.M.K.V.K.S). The order of the sessions was kept confidential until the end of the research and was known only to K.E.F., who was not involved in data collection.

A caffeine dosage of 5 milligrams per kilogram of body mass was used, as it has been found to be effective in previous studies [18,47,48] while presenting a relatively low incidence of side effects, which is essential to facilitate the blinding of the volunteer and researcher [22]. Both caffeine and placebo pills (pharmaceutical talc) were formulated in a compounding pharmacy. The formulated caffeine capsules contained 50 or 100 mg and were combined in order to achieve the desired supplementation dosage for different body masses as closely as possible (average dosage 5.0 mg/kg, range 4.6–5.3 mg/kg). Both the caffeine and placebo capsules had four different colors to ensure participants did not see the same color capsules during the experiment, helping blind them and the researcher performing the tests.

### 2.3. Procedures

In the first session, participants were familiarized with the tests and research procedures, completed a brief health history, performed anthropometric measurements, and were introduced to the questionnaires used by this study. After being introduced to the isokinetic equipment and ST test (1 maximal static contraction, 4 repetitions at three different speeds), they performed a Smith Machine Squat one-repetition maximum (1RM). The procedures for the muscular endurance test (ME) were then explained, followed by a brief familiarization. After the first session, participants’ body weight was reported to a different researcher who was responsible for preparing the correct dosage/pills for each participant according to the experimental session planned

After a minimum of 48 h from the first session, the familiarization session occurred. Upon arrival, participants answered the Perceived Recovery Status (PRS) scale, then performed another 1RM squat to confirm the previous value, and the ST and ME tests were performed for the first time. In the experimental sessions, participants received a placebo or caffeine (5 mg/kg of body weight) alongside information about the pill’s content according to the experimental design and then answered the Caffeine Side-Effects Questionnaire (CSE) four times every 15 min. The ST and ME tests were performed one hour after participants ingested the supplements. Capillary blood lactate was measured in the 2nd, 4th, and 6th minutes after the ME test. Blood lactate was only measured during the experimental sessions. 

### 2.4. Measurements

#### 2.4.1. Demographic Data and Questionnaires

In the first session, the researcher asked participants about their date of birth, duration, and frequency of strength training sessions, as well as the weekly frequency and duration of their physical activities (if any). Additionally, the researcher inquired whether the participants performed free squats or used a Smith machine and whether they engaged in any other physical activities, previous use of supplements, their perception of pre-workout supplements, their acute and chronic health status, smoking habits, sensitivity to caffeine, drug use, and any past use of anabolic steroids. After that, the researcher administered the Physical Activity Readiness Questionnaire (PAR-Q) [49] and asked participants about their dietary caffeine consumption. Participants were asked to maintain their habitual diet and caffeine consumption throughout the duration of the study.

Participants were also introduced to the Perceived Recovery Status (PRS) scale, with researchers explaining how to interpret the numerical and verbal anchors. The PRS was answered at the beginning of the control session and in every experimental session. Briefly, after warming up, participants were asked to estimate their recovery by indicating a number from 0 to 10, with a rating of ≥5 being associated with sufficient recovery [50].

Lastly, participants were introduced to the Caffeine Side-Effects Questionnaire (CSE). The CSE contains eight items in total, with seven being ergolytic effects of caffeine consumption (muscle soreness, diuresis, tachycardia/heart palpitations, anxiety, headache, gastrointestinal discomfort, and insomnia) and one ergogenic effect (increased vigor/activeness). When presented with the items, the participant verbally reported each item, indicating yes or no for each of the listed effects. The scale was used immediately after supplementation and every 15 min thereafter until one hour had passed.

#### 2.4.2. Anthropometry and Body Composition

Body mass was measured using an electronic scale with an accuracy of 0.5 kg (P 150M, Líder, Rio de Janeiro, Brazil) and height using a wall stadiometer with an accuracy of 0.1 mm (ES2020, Sanny, São Paulo, Brazil). Body composition was assessed using dual-energy X-ray absorptiometry (Lunar 8743 enCORE version 16, GE Health Care, Madison, WI, USA) on a separate day.

#### 2.4.3. Muscle Strength Testing Using an Isokinetic Dynamometer

To measure maximum strength (ST) in this study, an isokinetic dynamometer (System IV, Biodex Medical, Shirley, NY, USA) was used. The equipment was calibrated on every testing day in accordance with the manufacturer’s recommendations. Participants were adjusted to the equipment following the manufacturer’s guidelines. The participant was instructed to exert as much force as possible in the concentric phase of knee extension and not to exert force in the flexion phase. Participants performed a warm-up consisting of twelve repetitions at 180°/s, with researchers instructing them to gradually increase the force of contractions with the last three at maximal levels [51].

After the warm-up, ST testing began: two sets of one maximal isometric contraction at a knee flexion angle of 60° (0° being equivalent to a complete knee extension), two sets of four maximal voluntary contractions at 60°/s, 180°/s, 300°/s, with a two-minute interval between sets. All sets were performed on the right lower limb.

#### 2.4.4. Muscle Endurance Test

The muscular endurance (ME) protocol consisted of three sets of maximum repetitions performed until volitional fatigue, characterized by the inability to perform a concentric contraction. A 10–15 min interval was given between the ST and ME tests. Participants squatted using a Smith machine (guided bar) with a load of 60% 1RM [30,52] and a three-minute strictly controlled passive recovery between each set. The eccentric and concentric contraction phases were performed in two seconds each. The cadence was maintained using a digital metronome (MetronomeFree, Geppo Production, Tortolì, Italy) at a rate of 60 bpm. To help the subject perform the movement at the correct cadence, the researcher counted the movement times (one, two, three, four), informing the subject in advance that at “two” they should be at the end of the eccentric phase and at “four” they should finish the concentric phase of the movement.

In order to standardize the repetition range of motion, an elastic band was placed in the equipment so that participants would know when to start the concentric phase, limiting the knee flexion angle to approximately 80° (parallel squat). The position of the band was adjusted individually in the familiarization session for the 1RM test and was maintained in all following sessions. In our study, we opted to use three sets for the ME test, based on previous findings that caffeine may present its ergogenic effect only under fatiguing conditions [53,54].

#### 2.4.5. One-Repetition Maximum (1RM) Testing

The 1RM test protocol consisted of the following steps: an 8-repetition warm-up with an estimated load of 50% of the 1RM, followed by a one-minute rest, and then three repetitions with an estimated load of 70% of the 1RM. Finally, after another one-minute break, three to five attempts were performed to determine 1RM, with a five-minute break between them, with the value of the largest load used in the last successful attempt being recorded [51,55]. All 1RM values had less than 5% of variation between tests.

#### 2.4.6. Lactate Measurement

To analyze the potential influence of caffeine supplementation on anaerobic metabolism, blood lactate measurements were taken during the resting period before the tests and at the 2nd, 4th, and 6th minutes after the ME test. After aseptic cleaning, the earlobe was punctured with a disposable lancet. A volume of 25 μL (microliters) of arterialized capillary blood was obtained by using heparinized capillary tubes and then placed in Eppendorf tubes with 50 μL of 1% sodium fluoride for later analysis. Lactate concentrations (mmol/L) were measured using a lactate analyzer, model YSI 2300 Sport (Yellow Springs Inc., Yellow Springs, OH, USA), which operates by the electroenzymatic method. The analyzer was calibrated using a 5 mmol/L standard every ten measurements for greater precision, following the manufacturer’s recommendations. The YSI 2300 Sport has a reported coefficient of variation between 0.8 and 3.7% [56]. All exercise blood samples were analyzed at least twice to ensure precision. The average value was recorded. When the difference between measurements exceeded 0.5 mmol/L, a third dosage was performed (10.9% of 128 measurements).

### 2.5. Statistical Analysis

For statistical analysis, Microsoft Excel (version 14.0, Washington, DC, USA) software was used to record the data, and JASP (v. 0.19, Amsterdam, The Netherlands) was used for analysis. GraphPad Prism (v. 10.5, Boston, MA, USA) was used for graphing. Data were described in terms of mean and standard deviation. A 2 × 2 ANOVA (Informed × Received) was performed using the results from the ME test (total reps and session volume [reps × weight]), and lactate (2nd, 4th, and 6th minutes) as dependent variables. To analyze the effect of using and expecting caffeine on ST, we performed a 2 × 2 × 4 ANOVA for peak torque (Informed × Received × Contraction Speed) and a 2 × 2 × 3 ANOVA for maximal work (Informed × Received × Contraction Speed). A one-way ANOVA (one factor) was used to compare the resting lactate values. For post-hoc analyses and multiple T-tests, we employed Holm’s correction.

Following the rationale of the two-way ANOVA [37], we calculated for each individual caffeine’s effect using the sum of the results of the two sessions where the participant received caffeine minus the sum of the results from the two sessions where they received a placebo, divided by two (Equation (1)). Similarly, we calculated the effect of expecting to receive caffeine using the difference between the sum of the results of the two sessions where the participant was informed that they were receiving caffeine, minus the sum of the results from the two sessions where they were told they were receiving a placebo, divided by two (Equation (2)). We also calculated a relative change in caffeine usage and expectation effect using the results from Equations (1) and (2) as a percentage change in the individual’s performance in the control session. We analyzed whether caffeine’s effect and expectancy were significantly greater than zero using a single-sample T-test. Session acronyms in the equations are the same as Figure 1.

(1) $$\mathrm{Caffeine}’s\;\mathrm{effect}\;(\mathrm{EFF}):\;\frac{\left(C/C+P/C)-(C/P+P/P\right)}{2}$$

(2) $$\mathrm{Caffeine}’s\;\mathrm{expectancy}\;\mathrm{effect}\;(\mathrm{EXP}):\;\frac{\left(C/C+C/P)-(P/C+P/P\right)}{2}$$

Lastly, we explored the association between caffeine’s effect on ST and ME with caffeine consumption, body fat percentage, and relative maximal strength (1RM/body weight) by performing a Pearson correlation. We considered responders to caffeine to be those who showed an improvement in performance of >0%. When caffeine expectancy improved performance >0%, participants were considered as having a placebo response.

The significance level adopted for all analyses was *p* < 0.05. The strength of Pearson’s correlation coefficient was interpreted as small (r ≥ 0.1 and <0.3), moderate (r ≥ 0.3 and <0.5), large (r ≥ 0.5 and <0.7), very large (r ≥ 0.7 and <0.9), and extremely large (r ≥ 0.9) [57]. For pairwise comparisons, effect sizes were analyzed using Cohen’s “d” and interpreted as small (d ≥ 0.2 and <0.5), moderate (d ≥ 0.5 and <0.8), and large (d ≥ 0.8) [58].

## 3. Results

The participant characteristics are shown in Table 1. Participants had been resistance training for 2.8 ± 2.0 years, training 4 ± 1 days per week with a session duration of 54 ± 17 min. Caffeine consumption was very heterogeneous (range: 0 to 1880 mg/week); specifically, three participants consumed no caffeine during the week, and two consumed relatively higher amounts (>1750 mg/week). No participant reported physical recovery score (PRS) values lower than six on any day.

### 3.1. Strength Assessments—Peak Torque

**Figure 2:**
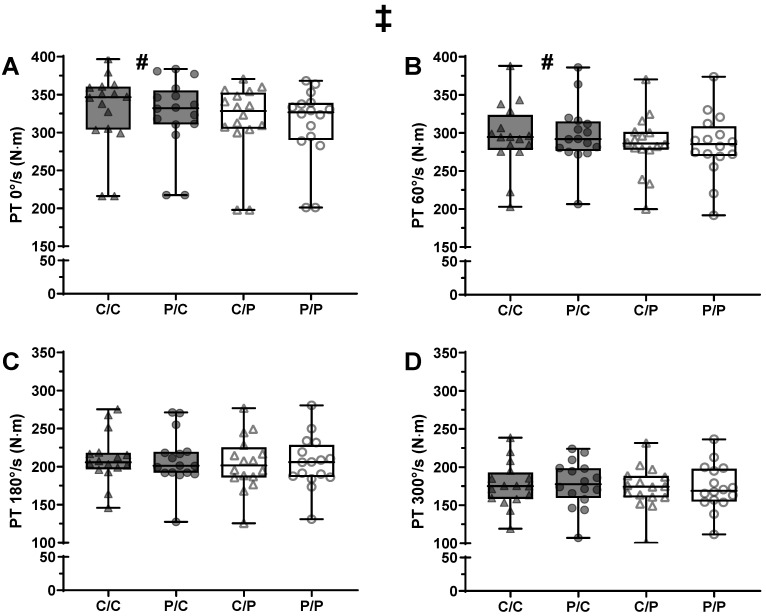
Box-plots of the peak torque (PT) achieved in the different experimental sessions across different contraction speeds in young, trained males (*n* = 16). Contraction speeds are 0°/s (**A**), 60°/s (**B**), 180°/s (**C**), 300 °/s (**D**). Triangles were used when participants were told they received caffeine, and circles when told that they received a placebo. Participants are represented by dark grey symbols when using caffeine, and clear symbols when using a placebo. Caffeine usage and contraction speed each had a significant main effect (*p* < 0.001 for both; no symbols shown). ‡: significant interaction between caffeine usage and contraction speed (*p* = 0.031). ^#^: significantly greater PT when using caffeine compared to a placebo, at each speed, as indicated by the symbol (*p* ≤ 0.003). C/C: told caffeine/used caffeine; P/C: told placebo/used caffeine; C/P: told caffeine/used placebo; P/P: told placebo/used placebo.

There was a significant main effect of caffeine usage on peak torque (PT) (*p* < 0.001) and contraction speed (*p* < 0.001) but not caffeine expectancy (*p* = 0.805; Figure 2). There was no interaction between caffeine usage and expectancy (*p* = 0.805). The only significant interaction term was between caffeine intake and contraction speed (*p* = 0.031). Post hoc analysis showed a small increase in peak torque (PT) with caffeine only during the static and 60°/s contraction speeds (*p* ≤ 0.001 & d = 0.3 for both), and not at 180°/s and 300°/s (*p* = 0.076–0.144; d = 0.1 for both).

### 3.2. Strength Assessments—Maximum Work

**Figure 3:**
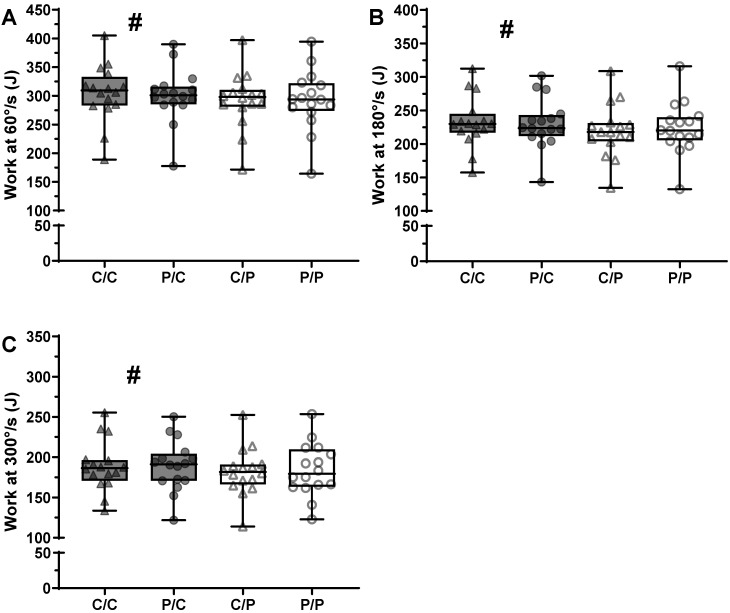
Box-plots of the maximum work (MW) performed in the different experimental sessions across different contraction speeds in young, trained males (*n* = 16). Contraction speeds are 60°/s (**A**), 180°/s (**B**), 300°/s (**C**). Triangles were used when participants were told they received caffeine, and circles when told that they received a placebo. Participants are represented by dark grey symbols when using caffeine, and clear symbols when using a placebo. Caffeine usage and contraction speed each had a significant main effect (*p* < 0.001 for both; no symbols shown). ^#^: significantly greater MW when using caffeine compared to a placebo, at each speed, as indicated by the symbol (*p* ≤ 0.018). C/C: told caffeine/used caffeine; P/C: told placebo/used caffeine; C/P: told caffeine/used placebo; P/P: told placebo/used placebo.

We observed significant main effects of caffeine and speed on maximum work (MW) (Figure 3; *p* < 0.001 for both), but not of being informed of receiving caffeine (*p* = 0.925). Unlike PT, no interactions were significant (*p* = 0.370–0.619), including caffeine intake and speed (*p* = 0.246). When considering all speeds combined, caffeine’s effect size was small (d = 0.2), which is somewhat consistent with the small effect sizes observed separately at 60°/s, 180°/s, and 300°/s—0.3, 0.2, and 0.1, respectively.

### 3.3. Muscle Endurance (ME)

**Figure 4:**
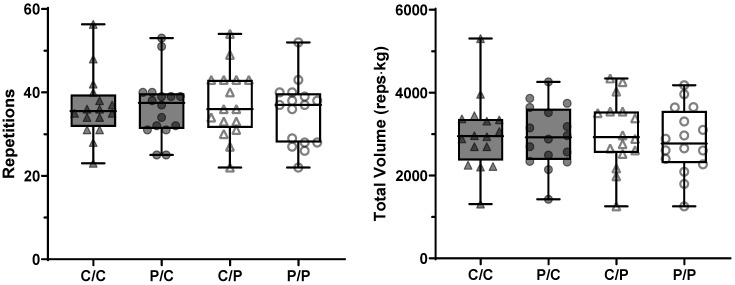
Box-plots of the muscle endurance test results (total number of repetitions, left panel, and the total volume of each experimental session, right panel) under differing caffeine (caffeine (C) vs. placebo (P)) and informed (C vs. P) conditions in young, trained males (*n* = 16). Triangles were used when participants were told they received caffeine, and circles when told that they received a placebo. Participants are represented by dark grey symbols when using caffeine, and clear symbols when using a placebo. No significant main effect or interaction, *p* ≥ 0.100. C/C: told caffeine/used caffeine; P/C: told placebo/used caffeine; C/P: told caffeine/used placebo; P/P: told placebo/used placebo.

We did not observe a significant effect of caffeine intake on total repetitions (*p* = 0.420) or total volume (*p* = 0.137, Figure 4). Caffeine’s expectancy also did not affect total repetitions (*p* = 0.194) nor total volume (*p* = 0.100). No significant interactions were observed in either analysis (*p* = 0.131 & *p* = 0.312). A summary of the study’s findings on PT, MW, and ME is shown in Table 2.

### 3.4. Blood Lactate Findings

**Figure 5:**
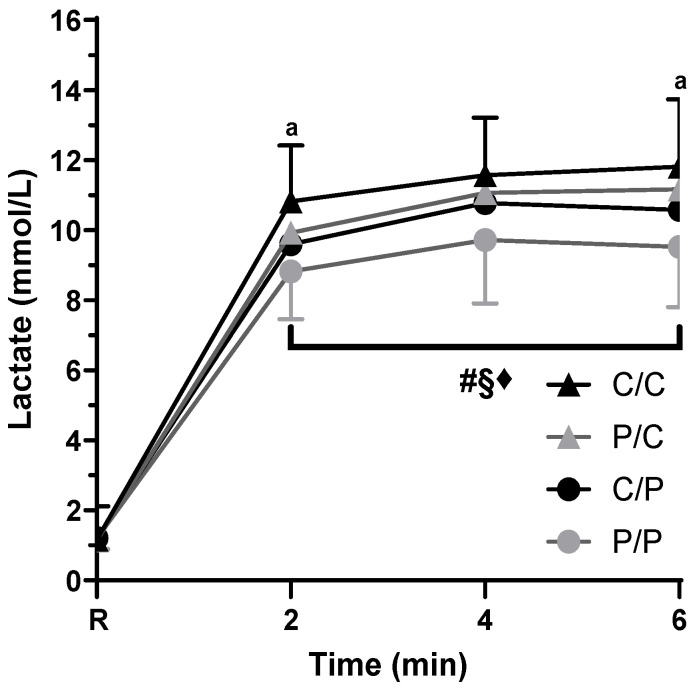
Blood lactate concentration at rest (R), 2nd, 4th, and 6th minutes after the muscle endurance test under differing caffeine (C v. P) and informed (C v. P) conditions in young, trained males (*n* = 16). Data are means ± SD. The # indicates a significant main effect of caffeine use on blood lactate values, § the significant effect of caffeine expectation, while the ♦ represents a significant time effect. No significant interactions detected (*p* = 0.139–0.964). a: Blood lactate values while using caffeine were significantly greater than when using a placebo at this time point.

Resting lactate values were not different between sessions (*p* = 0.768). Our analysis indicated a main effect of caffeine intake (*p* < 0.001), caffeine expectancy (*p* = 0.003), and time (*p* < 0.001) without any significant interaction between factors (*p* = 0.240–0.973) (Figure 5). Using caffeine resulted in a moderate increase in post-exercise blood lactate level (1.2 mmol/L) compared to placebo (d = 0.7), and being told they were given caffeine (caffeine expectancy) resulted in a small blood lactate increase of 0.8 mmol/L greater than when being told they were given a placebo (d = 0.4). Regarding time’s effect, lactate values were 1.0 mmol/L, moderately higher, at the sixth and fourth minute compared to the second minute (*p* ≤ 0.002; d = 0.6). However, there was no significant difference in lactate values between the fourth and sixth minutes (*p* = 0.813; d = 0.0). We also analyzed the effect of caffeine and its expectation at different time intervals. Following the main effects, using caffeine instead of a placebo resulted in moderately higher blood lactate levels at the 2nd minute (1.1 mmol/L, *p* = 0.018, d = 0.6) and significantly larger at the 6th minute (1.4 mmol/L, *p* = 0.003, d = 0.8). At the 4th minute, despite the moderate difference between using caffeine and a placebo, there was no statistical significance (1.0 mmol/L, *p* = 0.307, d = 0.5).

To explore the relationship between lactate values and the work performed during the sessions, we correlated lactate values with the number of repetitions and total training volume (Table 3). Significantly large correlations occurred only in sessions where participants were informed they were receiving caffeine (C/C and C/P), with no significant correlations in sessions where participants were told they were receiving a placebo (P/P), even when caffeine was administered (P/C).

### 3.5. Individual Caffeine Effect (EFF) on Muscle Strength and Endurance

To explore the variability in responses to caffeine supplementation, we calculated caffeine effect (EFF) for each individual. Findings are presented first as a group, then as the whole sample, and finally only among the responders. We present data first for PT, then for MW, and finally for ME. In line with the ANOVA findings, EFF was significantly greater than zero when analyzing PT at static and 60°/s, only (*p* < 0.001 and *p* = 0.012, respectively). The average relative EFF of caffeine on PT was 4.7 ± 5.5% (range: −3–16%), 4.2 ± 3.1% (range: −1–12%), 2.5 ± 4.6% (range: −7–10%), and 2.3 ± 5.3% (range: −7–11%) for static, 60°/s, 180°/s, 300°/s contraction speeds, respectively. Also, 69%, 88%, 69%, and 50% of individuals were considered caffeine responders for static, 60°/s, 180°/s, and 300°/s contraction speeds, respectively. Among them, the average caffeine EFF seemed to be superior to the sample average at 7.5 ± 4.3% (range: 1–16%), 4.9 ± 2.7% (range: 2–12%), 5.0 ± 2.9% (range: 2–10%), and 6.8 ± 2.7% (range: 3–11%) for static, 60°/s, 180°/s, 300°/s contraction speeds, respectively.

Regarding MW, the calculated EFF for caffeine was significantly greater than zero for the three speeds (*p* < 0.001—*p* = 0.015). For contraction speeds of 60°/s, 180°/s, and 300°/s, we observed 81%, 81%, and 63% of the sample as caffeine responders. The average relative EFF of caffeine on MW was 3.8 ± 3.3% (range: −1–11%), 4.4 ± 5.1% (range: −5–14%), and 3.2 ± 4.7% (range: −5–11%), for 60°/s, 180°/s, and 300°/s contraction speeds, respectively. Among responders, the average caffeine EFF seemed to be superior to the sample average, especially at faster speeds: 4.7 ± 3.0% (range: 1–11%), 6.0 ± 4.1% (range: 1–14%), 6.2 ± 2.7% (range: 1–11%) for 60°/s, 180°/s, 300°/s contraction speeds, respectively.

We also explored this variability in the ME response to caffeine. In line with the ANOVA findings, EFF on total repetitions was not significantly greater than zero (*p* = 0.367). However, some individuals (56%) were classified as caffeine responders. The average EFF in the entire sample was 2.3 ± 9.7% (range −12–14%). The average caffeine EFF seemed considerably higher in responders, with an average of 9.1 ± 3.3% (range 3–14%).

### 3.6. Individual Response to Caffeine Expectancy (EXP) on Muscle Strength and Endurance

To explore, if any, the variability in responses to caffeine expectancy, we analyzed its influence (EXP) individually. Only the responders’ findings are shown since no group effect was shown in the ANOVA. We present data first for PT, then for MW, and finally for ME. The EXP of caffeine on peak torque did not present a significant effect when examining the group as a whole (*p* = 0.527—*p* = 0.860). Still, some had a placebo effect and responded to caffeine expectancy, equivalent to 63%, 63%, 56%, and 50% of the group during static, 60°/s, 180°/s, and 300°/s contraction speeds, respectively. Among them, the average caffeine EXP was: 6.5 ± 2.5% (range: 2–13%), 4.9 ± 2.6% (range: 1–9%), 3.3 ± 2.6% (range: 1–8%), and 4.2 ± 3.2% (range: 1–11%) for static, 60°/s, 180°/s, 300°/s contraction speeds, respectively. Only 19% of the sample were consistently considered responders to caffeine EXP in PT at all speeds investigated.

Similar to PT, caffeine expectancy (EXP) on MW, on average, was statistically similar to zero (*p* = 0.737–0.815) but was heterogeneous, with sample responder rates of 63%, 50%, and 63% for contraction speeds of 60°/s, 180°/s, and 300°/s, respectively. Among them, the average caffeine EXP was 4.9 ± 2.4% (range: 1–9%), 4.0 ± 2.7% (range: 1–7%), and 3.3 ± 4.2% (range: 1–15%), for 60°/s, 180°/s, and 300°/s contraction speeds, respectively. Only 38% of the sample were consistently considered responders to caffeine EXP in MW at all speeds investigated.

Lastly, caffeine EXP did not influence total repetitions during the ME test (*p* = 0.292). Again, there were also individuals who responded to caffeine expectancy (63%), with an average relative change in performance due to caffeine EXP of 9.5 ± 6.6% (range 2–21%). A summary of findings discussed in Section 3.5 and Section 3.6 is presented in Table 4.

### 3.7. Exploratory Analyses and Correlations of Caffeine’s Effect

We analyzed responder rates in different contraction speeds for PT and MW and total repetitions for ME. We explored the consistency of responders in different tests and the frequency of caffeine responders and placebo responders (caffeine expectancy). First, for PT, only 38% of the sample consistently responded to caffeine across all four speeds investigated, whereas for MW, 50% of the sample were caffeine responders across all three speeds. To explore the relationship between caffeine’s effects on PT and MW, we performed multiple correlations between these variables (Table 5). It is interesting to note that, generally speaking, the correlations were stronger and more frequent between the “slower” contractions (static and 60°/s), while the faster speeds (180 and 300°/s) were mostly significantly correlated only with each other.

Regarding caffeine responders in ME, only 13% of the sample were responders to ME and PT (all speeds), while 31% were caffeine responders in both ME and the MW. Only 13% of the sample were caffeine responders based on the calculated EFF on ME, PT, and MW at all speeds investigated. The EFF of caffeine on ME was not correlated with its EFF on PT and MW at any speed (r = −0.18–0.29; *p* = 0.27–0.95).

We also analyzed possible overlap between EFF and EXP in the various variables. The rate of a participant being a responder to both caffeine EFF and EXP varied between 25–56% for PT, 44–50% for MW, and 31% for total repetitions. On PT, only one participant (6%) was a consistent responder considering EFF and EXP on all speeds analyzed. On MW, 19% were consistent responders considering EFF and EXP on all speeds analyzed. Only one participant (6%) was a consistent responder considering EFF and EXP on all variables analyzed (PT in all speeds, MW in all speeds, and total repetitions).

Lastly, we explored potential relationships between EFF and other variables. We performed multiple correlations, but the only significant correlation was a large negative correlation (r = −0.59; *p* = 0.017) between caffeine’s effect on ME (total repetitions) and relative maximum strength (1 RM/kg). All other correlations EFF on PT, EFF on ME, body mass index, percentage body fat, 1RM, caffeine consumption, training history in years, and minutes of resistance training session duration per week were moderate (negative and positive) to small, with non-significant *p*-values (*p* = 0.099–0.997). The self-reported incidence of caffeine side effects was low, precluding any statistical analysis. During the P/P and P/C sessions, there were two reports of side effects on each day. However, this was strikingly different when participants were informed that they had been given caffeine, with a total of 9 and 8 reports of side effects for sessions C/C and C/P, respectively.

## 4. Discussion

This study compared the effect of caffeine supplementation (5 mg/kg of body mass) on the maximum strength and muscular endurance of trained young men using a “balanced placebo design”. A secondary aim was to explore the individual variability in the responses to caffeine utilization and the placebo effect. Indeed, caffeine significantly increased strength; however, caffeine did not increase muscle endurance and caffeine expectancy did not affect both strength or muscle endurance. Surprisingly, blood lactate was affected by both caffeine use and expectancy. We confirmed the hypothesis that we would observe a significant variability in responses to caffeine and its expectancy. To our knowledge, this is the first investigation to address the effect of caffeine on strength and muscle endurance while exploring individual responses to caffeine and its expectancy (placebo effect) using a balanced placebo design.

### 4.1. Muscle Strength

We observed a significantly small effect of caffeine on strength when assessed by PT and MW (d = 0.2), which was approximately the same effect reported by various systematic reviews, which describe a small, significant increase in strength: 0.2 [3,4,10]. Interestingly, our data showed a significant interaction between the speed at which PT was investigated and caffeine’s effect. Although this interaction was not observed for MW, the observed effect size for MW appeared to be greater at 60°/s than at 180°/s and 300°/s, corroborating findings in PT (0.3 vs. 0.2 and 0.1, respectively). Our results contrast with previous research, which observed a significant effect of caffeine only at higher angular velocities [14,16]. However, our findings are supported by other studies, which have observed increases in static contraction peak torque and at slower angular speeds [9,13,15]. Another study using the same caffeine dose as ours did not observe a significant effect of caffeine on peak torque at 180°/s, a finding consistent with our results [18]. This highlights an important methodological consideration in exploring the effects of caffeine on muscle strength.

From a physiological standpoint, caffeine is normally associated with adenosine antagonism and increased nervous system activation, specifically: increased motor unit recruitment, increased muscle activation, increased neural drive to the muscles, and increased motoneuronal excitability [15,47,59,60]. However, recent work suggests that caffeine may also improve muscle function by acting directly on this tissue [3,61]. Interestingly, not only do several investigations in isolated muscle fibers from humans and animals report a direct effect of caffeine on the muscle, but they also report a greater caffeine effect on type I muscle fibers [62]. We propose this as a possible explanation for why caffeine seemed to have a different effect on muscle contractions performed at 180°/s and 300°/s and for the heterogeneity of caffeine responses [62,63,64]. Although this relationship between fiber type, contraction speed, and caffeine effect has not yet been directly investigated in humans, our results seem to support this hypothesis. If the effect of caffeine is lower in fast-twitch muscle fibers, caffeine would present a lower effect in activities that require greater participation of this type of fiber, such as contractions at 300°/s. It is known that there is an association between the predominance of fast muscle fibers and the torque or work produced at higher angular velocity contractions using an isokinetic dynamometer [65,66,67].

Similarly, the percentage of type I fiber distribution has been correlated to the torque produced at 60°/s [68]. The contribution of fast-twitch fibers to faster contraction speeds is also evident in intervention studies, which report greater hypertrophy of fast-twitch type fibers compared to slow-twitch fibers and an increase in the proportion of fast-twitch fibers after isokinetic training at higher angular velocities [69,70]. However, it is possible that caffeine improves physical function through more than one pathway, as we observed a similar magnitude of improvement in caffeine responders when examining faster and slower contraction velocities despite the seemingly lower frequency of responders at fast contraction speeds (averages of 7.5% and 4.9% for static and 60°/s, compared to 5.0% and 6.8% for 180°/s and 300°/s). Thus, alongside the genetic variation in caffeine metabolism [60,71], type I muscle fibers, which show a greater ergogenic response [62], could be one of the mechanisms that explain the large interindividual difference in caffeine response.

Based on our findings, we believe that the expectancy effect of caffeine on muscle strength is usually negligible; however, the expectancy effect improved PT and MW in some individuals to a similar magnitude as the average caffeine improvement. Interestingly, only a few of them experienced an additive effect of expectancy and caffeine’s physiological effects. This additive effect could help explain the discrepancy between caffeine’s small improvements seen in tightly controlled investigations, which report small effect sizes, and the perception of a small group of individuals who report caffeine as exerting significant changes [6,37]. Unlike our study, this additive effect could pose an obstacle to determining caffeine’s “true” effect in studies with very high dosages (≥9 mg/kg of body weight), where side effects would be very hard to mask caffeine usage.

Our results align with the most frequently observed findings in recent literature on the effect of caffeine on muscle strength. Studies using knee extensors generally observe significant effects of caffeine on strength [15,16,17,47]. Additionally, MW has recently been proposed as a more representative measure of muscle function than PT, as it represents the force exerted throughout the entire range of motion (Work = Force × Displacement) [72,73]. There is currently a lack of studies reporting data regarding caffeine and maximal isokinetic work.

To the best of our knowledge, there is only one other study that has investigated the effect of caffeine expectancy using a balanced placebo design and the same caffeine dosage. Similar to our findings, they observed a significant caffeine effect on PT, with CC and PC trials being greater than the PP trial, and caffeine expectancy did not impact strength [48]. In contrast to our findings, they did not observe any significant interaction between speeds (30 and 120°/s). This is likely due to the fact that in our study, four different contraction speeds were tested, rather than only two, thereby facilitating a more comprehensive comparison between “slower” and “faster” speeds. Unfortunately, no data on individual responses or lactate levels are reported, preventing us from performing a direct comparison with our own findings.

### 4.2. Muscle Endurance

Caffeine may enhance muscle endurance by increasing Na+/K+ ATPase activity (attenuates interstitial K+ accumulation), increasing calcium mobilization from the sarcoplasmic reticulum (higher release and/or lower reuptake) during contraction, and/or decreasing pain perception [1,43,60]. However, in the present study, we did not observe a significant effect of caffeine on muscle endurance, contradicting the findings of previous meta-analyses [3,12].

We were not alone in not observing a caffeine-based increase in muscular endurance [20,52,74]. However, there is no clear, significant methodological difference between studies that reported significant differences and those that did not, which helps explain the discrepancy in findings. Regarding methodological aspects that could affect ME, a meta-analysis examining muscle endurance during isotonic exercise reported that supplement timing was the only significant moderator of caffeine’s effect, with benefits observed only when caffeine was ingested 60 min before exercise; muscle group, dose, and form of ingestion were not significant moderators [12]. A separate meta-analysis similarly found no moderating effects of training experience, caffeine form, contraction type, or muscle group size/location, and reported that the only significant moderator was test type, with improvements observed only in open-endpoint protocols [3]. Dosages (5–6 mg/kg), population (trained individuals and athletes), and exercises performed (leg press and bench press) in the aforementioned studies [20,52,74] were similar to those in studies reporting performance-enhancing benefits of caffeine [2,21,54,75], yet the results differed.

Even within studies that observed positive effects of caffeine, inconsistencies exist. For example, Woolf et al. [20] reported that caffeine significantly increased total session volume (sets x repetitions x load) only during the bench press, not during the leg press. In alignment with the previously mentioned meta-analysis [12], muscle group does not seem to be a plausible explanation, as in direct opposition to the previous study, Green et al. [54] observed an increase in ME by caffeine only during the third leg press exercise set, with no improvement in any of the bench press sets. Contrary to the previous study, other investigations have observed a significant increase in the total number of repetitions when caffeine is used only in the first set of knee extension and squat exercises [2,21]—without any effect on the “biceps curl” exercise (elbow flexion) or on the bench press. Lastly, despite Astorino et al. [75] comparing four different exercises performed in this exact order—bench press, leg press, lat pull, and shoulder press—caffeine had an effect only on the leg press, significantly increasing the number of repetitions in the first and second sets out of four, but without a significant effect on total volume (reps x load). This begs the question as to what could be the potential explanation for the divergences in the results. This methodological and findings conundrum has not allowed for a precise mechanistic investigation, with the two main hypotheses on the variability of individual responses lying on the shoulders of the individual caffeine metabolic capacity (detailed in Section 4.4 of the discussion) and the inclusion of heavy caffeine users who suffer from withdrawal symptoms during placebo trials [1,23,75].

Our hypothesis is that previous studies [20,52,74] obtained results similar to ours, with samples containing a mix of responders and non-responders, the latter showing worse performance in the caffeine trials, resulting in zero net improvement. We had a responder rate (caffeine’s effect > 0) of 56% with an average caffeine effect on total repetitions of 9.1 ± 3.3% (range 3–14%). Few studies report the variability of responses and/or responder rates to caffeine supplementation, although it has been known for some time that caffeine’s effect on muscle endurance is heterogeneous [76]. There are cases in which 47% of participants improved their performance [21], 59% [74], 67% [20], and 72% [75]. It is intriguing to consider the actual performance of the remaining sample, that is, whether their performance did not improve or if it deteriorated.

Few studies have reported whether there was a worsening of performance with the use of caffeine and the number of cases, as the focus is usually on performance benefits. Hudson et al. [21] reported that half of the sample exhibited no improvement, and 13% experienced a decline in their performance, which, in their study, was defined as performing five more repetitions in the placebo session than in the caffeine session. Our findings, along with those of other studies reporting no average caffeine improvement in performance, may indicate that the presence of not only non-responders but also individuals whose performance worsens with caffeine. Thus, the balance between the number of individuals with both positive and negative responses to caffeine will determine if the overall effect in the sample is null.

### 4.3. Blood Lactate

Studies investigating caffeine’s effect on anaerobic performance and metabolism should be cautious when analyzing lactate data. Our analysis indicated that despite the lack of a significant increase in the average muscle endurance, caffeine had a moderate effect on lactate levels, particularly at the 6th minute after exercise (Figure 5). Additionally, caffeine expectancy had a small but significant effect on lactate levels. Thus, investigations attempting to tie increased high-intensity “anaerobic” exercise performance due to caffeine supplementation with lactate levels should be skeptical of a relationship between them, despite past attempts to do so [43]. For example, we observed significant correlations between lactate levels and repetitions or total volume only in sessions where the participant was informed that he was receiving caffeine (C/C and C/P) and not when they received caffeine without knowing it (P/C). Thus, although caffeine may increase “anaerobic performance” [4], it is unlikely that the main mechanism is increased muscle glycolytic flux, as proposed previously [43].

Regarding the mechanisms behind the increased lactate levels due to caffeine expectancy, the probable physiological pathway is related to anticipatory mechanisms of the central nervous system (CNS), resulting in increased sympathetic activity [77]. This interaction triggers a rise in catecholamine release, causes vasoconstriction and hypoperfusion, and boosts muscle tone and micro-contractions, which may explain the observed results [78,79]. For example, an investigation on lactate accumulation and psychosocial stress (simulated job interview followed by arithmetic operations) found a significant increase in blood lactate during stress compared to a non-stress control situation [80]. Thus, it is possible that caffeine expectancy generated an increase in sympathetic activity, which resulted in increased lactate production by non-exercising tissue, in accordance with previous research, where, despite an increase in arterial glucose and lactate, caffeine did not increase leg lactate release, muscle glycogenolysis, or muscle lactate concentrations [81]. Further mechanistic studies are necessary to better delineate the potential mechanisms and confirm this hypothesis.

### 4.4. Individual Variability

Currently, considerable scientific effort has been made toward investigating individual variability in caffeine response by assessing genetic differences associated with caffeine metabolism [23]. Despite a promising study comparing CYP1A2 polymorphisms and caffeine’s effect on endurance performance, these findings have not been successfully replicated, and research outside of endurance performance is lacking. Additionally, genotype variation related to the Adenosine A2A receptor expression (ADORA2A) has been shown to be associated with responses to caffeine in non-exercise scenarios, such as sleep disturbances and postprandial glycaemia [24]. Adenosine A2A receptor density could help explain the differences in response to caffeine between trained and untrained individuals, as endurance-trained athletes present a greater density of these receptors [42,82]. Unfortunately, research investigating these genetic variations in relation to strength and muscle endurance is sparse and yields conflicting results [83].

Other variables commonly proposed to interfere with caffeine response include habitual caffeine intake, body composition, and training status [60,71]. In our study, none of these variables were correlated with the effect on PT, MW, or repetitions, except for a large negative correlation between caffeine’s effect on the total number of repetitions and 1RM/BW. The 1RM/BW ratio has been proposed as a complementary measure to assess training status, rather than relying solely on current uninterrupted training time. Authors have used ratios between 1.3 and 1.5 to classify individuals as advanced lifters [84]. Following our correlational finding, we observed that all individuals with a 1RM/BW >1.8 exhibited a performance decrement using caffeine (EFF < 0), while all individuals with a 1RM/BW <1.5 were responders (EFF > 0). While it is unlikely that being highly trained negates caffeine’s effect, such findings underscore the need for further research not only on genetic predisposition but also on basic characteristics whose association with caffeine response remains equivocal.

Interestingly, in our study, only very few individuals were caffeine “responders” in both strength and muscle endurance testing. We hypothesize that the heterogeneous response to caffeine supplementation on muscle strength and endurance could be explained by its multiple sites of action. For example, one could respond to caffeine by showcasing increased torque production and muscle work due to increased muscle activation and other nervous system-related mechanisms, while not enhancing muscle endurance because of a lack of increase in sarcoplasmic calcium mobilization and Na+/K+ ATPase activity (peripheral mechanisms). Thus, we hypothesize that being a caffeine responder could be a result of a complex balance between the speed of caffeine metabolization, adenosine receptor density, training status, and caffeine habituation. New studies should also investigate the association between muscle fiber typing and caffeine response, as a significant gap exists between in vivo data on this topic and the substantial body of research on isolated muscle fibers that supports the hypothesis of a fiber-type-dependent caffeine response.

Thus, balancing all of the above with the correct caffeine dosage and timing could help us understand how to optimize caffeine usage to increase muscle strength and endurance, as some individuals have exhibited significant improvements. In contrast, others have shown small improvements or even performance decrements. Additionally, in most cases in our study, there was no overlap between the placebo effect and caffeine response; therefore, it is unknown whether, in a real-world scenario, it would be possible to combine both for optimal use of this substance as a performance-enhancing supplement.

### 4.5. Limitations

The isokinetic dynamometer is a gold-standard, reliable measure of strength. However, it is unknown how much the small effect size observed translates to complex exercise and sports tasks. Contrary to the strength assessment, the muscle endurance test is closer to a “real-world” task, resembling closely what may happen in a resistance training session (external validity). However, the lack of reliability data on this type of testing limits our analysis. It is unknown what percentage of improvement or worsening of performance exceeds the test’s natural variability and is clinically meaningful.

In assessing the individual effects of caffeine, it is noteworthy that the variability in response to caffeine surpassed our expectations. Furthermore, our study is among the few reports of a performance decline associated with caffeine supplementation. This unforeseen result, coupled with the sample size, may have hindered our analyses between responders and non-responders, despite our study exceeding the targeted sample size for the primary analysis. Also, we did not restrict caffeine consumption (e.g., coffee, energy drinks, chocolate) and used a caffeine supplementation approach in which habitual dietary habits were maintained, and caffeine supplementation exceeded habitual intake. Thus, our findings might have differed if participants had been instructed to restrict caffeine consumption.

Participants were trained young resistance-trained men; therefore, we are unable to extrapolate our results to women, older men, or untrained men. Similarly, our findings cannot be extrapolated to all muscle groups or exercises, as strength parameters were assessed only in the knee extensor muscles, and endurance was assessed using squats. Lastly, while participants had >2 days between experimental sessions, with an average of 5 days, there was some variability in time between sessions, and we cannot exclude the possibility that modest differences in time between sessions could alter the present findings.

Our study employed a non-random convenience sampling method, which may introduce bias into our findings, particularly regarding past experiences and expectations with caffeine supplementation. This sampling approach and our recruitment strategy are known to be associated with self-selection bias and motivation bias; for example, we may have oversampled individuals who had positive experiences with this supplement, who are highly motivated to exercise, and involuntarily did not include in the research those who had neutral or negative experiences with caffeine. Additionally, our findings are influenced by the healthy volunteer effect; for instance, caffeine’s adverse effects are likely underestimated because potential participants are less inclined to enroll if they anticipate negative reactions during the supplementation protocol.

### 4.6. Future Research Directions

Considering our findings, future studies should investigate whether the predominance of a particular muscle fiber type may be one of the factors explaining the heterogeneity of caffeine responses. Thus, new studies investigating variability (e.g., fiber type dependence or responders/non-responders) and mechanisms should plan for larger studies and, whenever possible, include a test-reliability measure within the study to distinguish small effects from test variability.

## 5. Conclusions

This study is the first to investigate the effects of caffeine supplementation and expectancy on muscle strength and endurance in young resistance-trained men, using a double-blind, balanced placebo design. Caffeine supplementation showed a significant effect on muscle strength that superseded the placebo effect, which was negligible. This is an encouraging finding, as if the placebo effect were significant, it would be highly recommended that future research involving caffeine employ a balanced placebo design rather than the conventional double-blind design.

For muscle endurance, on the other hand, neither caffeine nor its expectancy increased muscle endurance, even though blood lactate levels rose due to both caffeine intake and expectancy. These findings suggest that caffeine does not affect muscle anaerobic glycolysis, as the significant increase in blood lactate was not accompanied by an increase in work performed during the test. Interestingly, approximately half of the participants improved their muscle endurance, while the rest worsened their performance. Thus, although caffeine’s main site of action is the nervous system, we hypothesize that other physiological mechanisms may operate in parallel, leading to significant inter-individual variability in the muscle endurance response, as evidenced by inconsistent responses to the strength and ME tests. In other words, because caffeine responders in the ME test were not consistently responders in the strength tests, we hypothesize that variability in responses is not solely attributable to central mechanisms of caffeine ergogenicity. In our study, these heterogeneous responses in ME were not correlated with habitual caffeine intake, body composition, or increased muscle strength from caffeine supplementation. Further research is necessary to elucidate possible mechanisms, especially the hypothetical role of fiber-type predominance and its influence on the variability of the caffeine response.

Lastly, as a practical application of our findings for athletes and coaches, we highlight that caffeine supplementation of 5 mg/kg increased muscle strength. They should also be aware that most of the time, improvements are modest. Coaches and athletes planning to use caffeine to enhance lower-body muscle endurance should carefully tailor the supplementation protocol, as our study observed that caffeine increased performance in some individuals but decreased it in others. Our findings are limited to the characteristics of our study sample and cannot necessarily be extrapolated to women, older men, and non-resistance-trained individuals.

## Figures and Tables

**Table 1:** Descriptive characteristics of the participants (*n* = 16).

	Age (Years)	BW (kg)	Height (cm)	%BF * (%)	CAF/w (mg/Week)	1RM (kg)	1RM/BW
Average	21.4	81.8	177.4	17.6	657.2	134.7	1.7
SD	2.9	11.1	6.2	4.0	617.2	24.1	0.3

Data are expressed as mean ± standard deviation. Abbreviations: SD: Standard deviation; BW: Body weight; %BF: Body fat percentage; CAF/w: Weekly caffeine consumption; 1RM: 1 repetition maximum. * *n* = 14.

**Table 2:** Summary of the main study outcomes (means ± SD) and findings in a sample of young, trained males (n = 16) under different caffeine use and informed conditions.

Outcomes	Condition C/C	Condition C/P	Condition P/C	Condition P/P	Summary of the Findings
Static PT (N∙m)	327 ± 55	313 ± 58	323 ± 53	310 ± 54	↑PT when using CAF (d = 0.3)
PT at 60°/s (N∙m)	296 ± 46	285 ± 43	296 ± 45	283 ± 46	↑PT when using CAF (d = 0.3)
PT at 180°/s (N∙m)	211 ± 33	204 ± 36	209 ± 35	206 ± 34	↔PT when using CAF (d = 0.1)
PT at 300°/s (N∙m)	177 ± 29	173 ± 28	178 ± 30	174 ± 30	↔PT when using CAF (d = 0.0)
MW at 60°/s (J)	305 ± 50	292 ± 49	301 ± 47	293 ± 52	↑MW when using CAF (d = 0.3)
MW at 180°/s (J)	232 ± 38	220 ± 40	230 ± 37	242 ± 39	↑MW when using CAF (d = 0.2)
MW at 300°/s (J)	189 ± 31	182 ± 30	189 ± 32	184 ± 33	↑MW when using CAF (d = 0.1)
Total reps.	37 ± 10	37 ± 8	37 ± 8	35 ± 8	↔reps when using CAF (d = 0.1)
Total vol. (reps∙kg)	2998 ± 881	3025 ± 847	2949 ± 741	2836 ± 795	↔total vol. when using CAF (d = 0.1)

C/C: told caffeine/used caffeine; P/C: told placebo/used caffeine; C/P: told caffeine/used placebo; P/P: told placebo/used placebo; PT: peak torque; ↑: statistically significant increase (*p* < 0.05); CAF: caffeine; d = Cohen’s d for using caffeine vs. placebo; ↔: lack of statistically significant difference between conditions (*p* > 0.05); MW: maximum work; Reps: repetitions; Vol: volume.

**Table 3:** Pearson correlation (r) between blood lactate values, total repetitions, and total volume lifted after the muscle endurance test in young, trained males (*n* = 16) under different caffeine use and informed conditions.

Session	Variable	Lac2			Lac4			Lac6	
		R	*p*		R	*p*		R	*p*
C/C	Repetitions	0.55	0.03 *		0.48	0.06		0.42	0.11
	Total Volume	0.59	0.02 *		0.53	0.04 *		0.35	0.18
C/P	Repetitions	0.52	0.04 *		0.47	0.06		0.57	0.02 *
	Total Volume	0.57	0.02 *		0.67	0.01 *		0.63	0.01 *
P/C	Repetitions	−0.02	0.93		0.49	0.06		0.38	0.15
	Total Volume	0.23	0.38		0.41	0.12		0.42	0.10
P/P	Repetitions	0.19	0.48		0.19	0.49		0.29	0.28
	Total Volume	0.33	0.22		0.36	0.17		0.19	0.49

Lac2: lactate in the 2nd minute; Lac4: lactate in the 4th minute; Lac6: lactate in the 6th minute of recovery; *: significant correlation (*p* < 0.05).

**Table 4:** Summary of the findings on individual variability in response to caffeine effect and caffeine expectancy (calculations explained in the methods) for peak torque (PT), maximal work (MW), and muscle endurance (ME).

		**Entire Sample (*n* = 16)**				**Responders Only**		
**Outcome**	**Speed**	**CAF EFF** **Mean ± SD**	**CAF EFF** **Range**	**EFF ≠ 0** **(*p*-Value)**		**Sample %**	**CAF EFF** **Mean ± SD**	**CAF EFF** **Range**
PT (N∙m)	Static	4.7 ± 5.5%	−3–16%	<0.001		69	7.5 ± 4.3%	1–16%
PT (N∙m)	60°/s	4.2 ± 3.1%	−1–12%	0.012		88	4.9 ± 2.7%	2–12%
PT (N∙m)	180°/s	2.5 ± 4.6%	−7–10%	0.088		69	5.0 ± 2.9%	2–10%
PT (N∙m)	300°/s	2.3 ± 5.3%	−7–11%	0.097		50	6.8 ± 2.7%	3–11%
MW (J)	60°/s	3.8 ± 3.3%	−1–11%	<0.001		81	4.7 ± 3.0%	1–16%
MW (J)	180°/s	4.4 ± 5.1%	−5–14%	0.006		81	6.0 ± 4.1%	1–14%
MW (J)	300°/s	3.2 ± 4.7%	−5–11%	0.015		63	6.2 ± 2.7%	1–11%
ME (total reps)	—	2.3 ± 9.7%	−12–14%	0.367		56	9.1 ± 3.3%	3–14%
**Outcome**	**Speed**	**CAF EXP** **Mean ± SD**	**CAF EXP** **Range**	**EXP ≠ 0** **(*p*-Value)**		**Sample %**	**CAF EXP** **Mean ± SD**	**CAF EXP** **Range**
PT (N∙m)	Static	—	—	—		63	6.5 ± 2.5%	2–13%
PT (N∙m)	60°/s	—	—	—		63	4.9 ± 2.6%	1–9%
PT (N∙m)	180°/s	—	—	—		56	3.3 ± 2.6%	1–8%
PT (N∙m)	300°/s	—	—	—		50	4.2 ± 3.2%	1–11%
MW (J)	60°/s	—	—	—		63	4.9 ± 2.4%	1–9%
MW (J)	180°/s	—	—	—		50	4.0 ± 2.7%	1–7%
MW (J)	300°/s	—	—	—		63	3.3 ± 4.2%	1–15%
ME (total reps)	—	—	—	—		63	9.5 ± 6.6%	2–21%

Data are expressed as mean ± standard deviation and range for relative percent change. Responders were defined as >0% change. CAF: caffeine; EFF: caffeine usage effect; PT: peak torque; MW: maximal work; ME: muscle endurance; EXP: caffeine expectancy effect.

**Table 5:** Pearson correlation matrix (r) between caffeine’s effect (EFF) in strength (via peak torque and maximum work) in young, trained males (*n* = 16). EFF was calculated as explained in Section 2.5.

	Speed (°/s)	PT				MW		
		0	60°/s	180°/s	300°/s	60°/s	180°/s	300°/s
PT	0	1						
	60	0.60 *	1					
	180	0.49	0.39	1				
	300	−0.04	−0.05	0.39	1			
MW	60	0.58 *	0.73 *	0.26	−0.14	1		
	180	0.57 *	0.34	0.85 *	0.52 *	0.48	1	
	300	0.19	0.23	0.67 *	0.90 *	0.08	0.74 *	1

PT: peak torque; MW: maximum work performed; *: statistically significant *p* < 0.05.

## Data Availability

The original contributions presented in the study are included in the article, further inquiries can be directed to the corresponding author.

## References

[1] Astorino T.A., Roberson D.W. (2010). Efficacy of Acute Caffeine Ingestion for Short-Term High-Intensity Exercise Performance: A Systematic Review. J. Strength Cond. Res..

[2] Karayigit R., Naderi A., Akca F., da Cruz C.J.G., Sarshin A., Yasli B.C., Ersoz G., Kaviani M. (2020). Effects of Different Doses of Caffeinated Coffee on Muscular Endurance, Cognitive Performance, and Cardiac Autonomic Modulation in Caffeine Naive Female Athletes. Nutrients.

[3] Warren G.L., Park N.D., Maresca R.D., McKibans K.I., Millard-Stafford M.L. (2010). Effect of Caffeine Ingestion on Muscular Strength and Endurance: A Meta-Analysis. Med. Sci. Sports Exerc..

[4] Grgic J., Grgic I., Pickering C., Schoenfeld B.J., Bishop D.J., Pedisic Z. (2020). Wake up and Smell the Coffee: Caffeine Supplementation and Exercise Performance-an Umbrella Review of 21 Published Meta-Analyses. Br. J. Sports Med..

[5] Xue R., Huang J., Chen B., Ding L., Guo L., Cao Y., Girard O. (2025). Effects of Caffeine Dose and Administration Method on Time-Trial Performance: A Systematic Review and Network Meta-Analysis. Nutrients.

[6] Del Coso J., Muñoz G., Muñoz-Guerra J. (2011). Prevalence of Caffeine Use in Elite Athletes Following Its Removal from the World Anti-Doping Agency List of Banned Substances. Appl. Physiol. Nutr. Metab..

[7] Kreutzer A., Graybeal A.J., Moss K., Braun-Trocchio R., Shah M. (2022). Caffeine Supplementation Strategies Among Endurance Athletes. Front. Sports Act. Living.

[8] Tallis J., Clarke N., Morris R., Richardson D., Ellis M., Eyre E., Duncan M., Noon M. (2021). The Prevalence and Practices of Caffeine Use as an Ergogenic Aid in English Professional Soccer. Biol. Sport.

[9] Scapec B., Grgic J., Varovic D., Mikulic P. (2024). Caffeine, but Not Paracetamol (Acetaminophen), Enhances Muscular Endurance, Strength, and Power. J. Int. Soc. Sports Nutr..

[10] Grgic J., Trexler E.T., Lazinica B., Pedisic Z. (2018). Effects of Caffeine Intake on Muscle Strength and Power: A Systematic Review and Meta-Analysis. J. Int. Soc. Sports Nutr..

[11] Goldstein E.R., Ziegenfuss T., Kalman D., Kreider R., Campbell B., Wilborn C., Taylor L., Willoughby D., Stout J., Graves B.S. (2010). International Society of Sports Nutrition Position Stand: Caffeine and Performance. J. Int. Soc. Sports Nutr..

[12] Polito M.D., Souza D.B., Casonatto J., Farinatti P. (2016). Acute Effect of Caffeine Consumption on Isotonic Muscular Strength and Endurance: A Systematic Review and Meta-Analysis. Sci. Sports.

[13] Venier S., Grgic J., Mikulic P. (2019). Caffeinated Gel Ingestion Enhances Jump Performance, Muscle Strength, and Power in Trained Men. Nutrients.

[14] Tallis J., Yavuz H.C.M. (2018). The Effects of Low and Moderate Doses of Caffeine Supplementation on Upper and Lower Body Maximal Voluntary Concentric and Eccentric Muscle Force. Appl. Physiol. Nutr. Metab. Physiol. Appl. Nutr. Metab..

[15] Behrens M., Mau-Moeller A., Weippert M., Fuhrmann J., Wegner K., Skripitz R., Bader R., Bruhn S. (2015). Caffeine-Induced Increase in Voluntary Activation and Strength of the Quadriceps Muscle during Isometric, Concentric and Eccentric Contractions. Sci. Rep..

[16] Duncan M.J., Thake C.D., Downs P.J. (2014). Effect of Caffeine Ingestion on Torque and Muscle Activity during Resistance Exercise in Men. Muscle Nerve.

[17] Timmins T.D., Saunders D.H. (2014). Effect of Caffeine Ingestion on Maximal Voluntary Contraction Strength in Upper- and Lower-Body Muscle Groups. J. Strength Cond. Res..

[18] Astorino T.A., Terzi M.N., Roberson D.W., Burnett T.R. (2010). Effect of Two Doses of Caffeine on Muscular Function during Isokinetic Exercise. Med. Sci. Sports Exerc..

[19] Trevino M.A., Coburn J.W., Brown L.E., Judelson D.A., Malek M.H. (2015). Acute Effects of Caffeine on Strength and Muscle Activation of the Elbow Flexors. J. Strength Cond. Res..

[20] Woolf K., Bidwell W.K., Carlson A.G. (2008). The Effect of Caffeine as an Ergogenic Aid in Anaerobic Exercise. Int. J. Sport Nutr. Exerc. Metab..

[21] Hudson G.M., Green J.M., Bishop P.A., Richardson M.T. (2008). Effects of Caffeine and Aspirin on Light Resistance Training Performance, Perceived Exertion, and Pain Perception. J. Strength Cond. Res. Natl. Strength Cond. Assoc..

[22] Pallarés J.G., Fernández-Elías V.E., Ortega J.F., Muñoz G., Muñoz-Guerra J., Mora-Rodríguez R. (2013). Neuromuscular Responses to Incremental Caffeine Doses: Performance and Side Effects. Med. Sci. Sports Exerc..

[23] Tallis J., Guimaraes-Ferreira L., Clarke N.D. (2022). Not Another Caffeine Effect on Sports Performance Study—Nothing New or More to Do?. Nutrients.

[24] Banks N.F., Tomko P.M., Colquhoun R.J., Muddle T.W.D., Emerson S.R., Jenkins N.D.M. (2019). Genetic Polymorphisms in ADORA2A and CYP1A2 Influence Caffeine’s Effect on Postprandial Glycaemia. Sci. Rep..

[25] Benedetti F. (2013). Placebo and the New Physiology of the Doctor-Patient Relationship. Physiol. Rev..

[26] Bérdi M., Köteles F., Szabó A., Bárdos G. (2011). Placebo Effects in Sport and Exercise: A Meta-Analysis. Eur. J. Ment. Health.

[27] Doherty M., Smith P.M. (2005). Effects of Caffeine Ingestion on Rating of Perceived Exertion during and after Exercise: A Meta-Analysis. Scand. J. Med. Sci. Sports.

[28] Beedie C.J. (2010). All in the Mind? Pain, Placebo Effect, and Ergogenic Effect of Caffeine in Sports Performance. Open Access J. Sports Med..

[29] Pollo A., Carlino E., Benedetti F. (2008). The Top-down Influence of Ergogenic Placebos on Muscle Work and Fatigue. Eur. J. Neurosci..

[30] Duncan M.J., Lyons M., Hankey J. (2009). Placebo Effects of Caffeine on Short-Term Resistance Exercise to Failure. Int. J. Sports Physiol. Perform..

[31] Beedie C.J., Foad A.J. (2009). The Placebo Effect in Sports Performance: A Brief Review. Sports Med..

[32] McClung M., Collins D. (2007). “Because I Know It Will!”: Placebo Effects of an Ergogenic Aid on Athletic Performance. J. Sport Exerc. Psychol..

[33] Pollo A., Amanzio M., Arslanian A., Casadio C., Maggi G., Benedetti F. (2001). Response Expectancies in Placebo Analgesia and Their Clinical Relevance. Pain.

[34] Montalvo-Alonso J.J., Del Val-Manzano M., Cerezo-Telléz E., Ferragut C., Valadés D., Rodríguez-Falces J., Pérez-López A. (2025). Acute Caffeine Intake Improves Muscular Strength, Power, and Endurance Performance, Reversing the Time-of-Day Effect Regardless of Muscle Activation Level in Resistance-Trained Males: A Randomized Controlled Trial. Eur. J. Appl. Physiol..

[35] Filip-Stachnik A., Krzysztofik M., Del Coso J., Wilk M. (2021). Acute Effects of High Doses of Caffeine on Bar Velocity during the Bench Press Throw in Athletes Habituated to Caffeine: A Randomized, Double-Blind and Crossover Study. J. Clin. Med..

[36] Saunders B., de Oliveira L.F., da Silva R.P., de Salles Painelli V., Gonçalves L.S., Yamaguchi G., Mutti T., Maciel E., Roschel H., Artioli G.G. (2017). Placebo in Sports Nutrition: A Proof-of-Principle Study Involving Caffeine Supplementation. Scand. J. Med. Sci. Sports.

[37] Foad A.J., Beedie C.J., Coleman D.A. (2008). Pharmacological and Psychological Effects of Caffeine Ingestion in 40-Km Cycling Performance. Med. Sci. Sports Exerc..

[38] Zagatto A.M., Lopes V.H.F., Dutra Y.M., de Poli R.A.B., Dolan E., Rasica L., Murias J.M., de Azevedo P.H.S.M. (2024). Sodium Bicarbonate Induces Alkalosis, but Improves High-Intensity Cycling Performance Only When Participants Expect a Beneficial Effect: A Placebo and Nocebo Study. Eur. J. Appl. Physiol..

[39] de Oliveira L.F., Dolan E., Swinton P.A., Durkalec-Michalski K., Artioli G.G., McNaughton L.R., Saunders B. (2022). Extracellular Buffering Supplements to Improve Exercise Capacity and Performance: A Comprehensive Systematic Review and Meta-Analysis. Sports Med..

[40] Hurst P., Schipof-Godart L., Hettinga F., Roelands B., Beedie C. (2020). Improved 1000-m Running Performance and Pacing Strategy With Caffeine and Placebo: A Balanced Placebo Design Study. Int. J. Sports Physiol. Perform..

[41] Vega-Muñoz A., Contreras-Barraza N., Salazar-Sepúlveda G., Lay N., Gil-Marín M., Muñoz-Urtubia N. (2024). Caffeine Placebo Effect in Sport and Exercise: A Systematic Review. Nutrients.

[42] Heinonen I., Nesterov S.V., Liukko K., Kemppainen J., Någren K., Luotolahti M., Virsu P., Oikonen V., Nuutila P., Kujala U.M. (2008). Myocardial Blood Flow and Adenosine A2A Receptor Density in Endurance Athletes and Untrained Men. J. Physiol..

[43] Davis J.K., Green J.M. (2009). Caffeine and Anaerobic Performance: Ergogenic Value and Mechanisms of Action. Sports Med..

[44] Mielgo-Ayuso J., Marques-Jiménez D., Refoyo I., Del Coso J., León-Guereño P., Calleja-González J. (2019). Effect of Caffeine Supplementation on Sports Performance Based on Differences Between Sexes: A Systematic Review. Nutrients.

[45] FIOCRUZ (2025). Manual do Registrante.

[46] Agência Nacional de Vigilância Sanitária (ANVISA) (2020). Resolução RDC n^o^ 449, de 15 de Dezembro de 2020.

[47] Black C.D., Waddell D.E., Gonglach A.R. (2015). Caffeine’s Ergogenic Effects on Cycling: Neuromuscular and Perceptual Factors. Med. Sci. Sports Exerc..

[48] Tallis J., Muhammad B., Islam M., Duncan M.J. (2016). Placebo Effects of Caffeine on Maximal Voluntary Concentric Force of the Knee Flexors and Extensors. Muscle Nerve.

[49] Shephard R.J. (2015). Qualified Fitness and Exercise as Professionals and Exercise Prescription: Evolution of the PAR-Q and Canadian Aerobic Fitness Test. J. Phys. Act. Health.

[50] Laurent C.M., Green J.M., Bishop P.A., Sjökvist J., Schumacker R.E., Richardson M.T., Curtner-Smith M. (2011). A Practical Approach to Monitoring Recovery: Development of a Perceived Recovery Status Scale. J. Strength Cond. Res. Natl. Strength Cond. Assoc..

[51] Brown L.E., Weir J.P. (2001). ASEP Procedures Recommendation I: Accurate Assessment of Muscular Strength and Power. J. Exerc. Hysiology Online.

[52] Astorino T.A., Rohmann R.L., Firth K. (2008). Effect of Caffeine Ingestion on One-Repetition Maximum Muscular Strength. Eur. J. Appl. Physiol..

[53] Meyers B.M., Cafarelli E. (2005). Caffeine Increases Time to Fatigue by Maintaining Force and Not by Altering Firing Rates during Submaximal Isometric Contractions. J. Appl. Physiol..

[54] Green J.M., Wickwire P.J., McLester J.R., Gendle S., Hudson G., Pritchett R.C., Laurent C.M. (2007). Effects of Caffeine on Repetitions to Failure and Ratings of Perceived Exertion during Resistance Training. Int. J. Sports Physiol. Perform..

[55] Martorelli A., Bottaro M., Vieira A., Rocha-Júnior V., Cadore E., Prestes J., Wagner D., Martorelli S. (2015). Neuromuscular and Blood Lactate Responses to Squat Power Training with Different Rest Intervals between Sets. J. Sports Sci. Med..

[56] Davison R.C., Coleman D., Balmer J., Nunn M., Theakston S., Burrows M., Bird S. (2000). Assessment of Blood Lactate: Practical Evaluation of the Biosen 5030 Lactate Analyzer. Med. Sci. Sports Exerc..

[57] Bradley P.S. (2024). “Setting the Benchmark” Part 1: The Contextualised Physical Demands of Positional Roles in the FIFA World Cup Qatar 2022. Biol. Sport.

[58] Cohen J. (1992). A Power Primer. Psychol. Bull..

[59] Behrens M., Mau-Moeller A., Heise S., Skripitz R., Bader R., Bruhn S. (2015). Alteration in Neuromuscular Function of the Plantar Flexors Following Caffeine Ingestion. Scand. J. Med. Sci. Sports.

[60] Soares E.d.M.K.V.K., Garcia G.L., Molina G.E., Fontana K.E. (2019). Muscle Strength and Caffeine Supplementation: Are We Doing More of the Same?. Rev. Bras. Med. Esporte.

[61] Tarnopolsky M., Cupido C. (2000). Caffeine Potentiates Low Frequency Skeletal Muscle Force in Habitual and Nonhabitual Caffeine Consumers. J. Appl. Physiol..

[62] Tallis J., Duncan M.J., James R.S. (2015). What Can Isolated Skeletal Muscle Experiments Tell Us about the Effects of Caffeine on Exercise Performance?. Br. J. Pharmacol..

[63] Takagi R., Tabuchi A., Hayakawa K., Osana S., Yabuta H., Hoshino D., Poole D.C., Kano Y. (2023). Chronic Repetitive Cooling and Caffeine-Induced Intracellular Ca^2+^ Elevation Differentially Impact Adaptations in Slow- and Fast-Twitch Rat Skeletal Muscles. Am. J. Physiol. Regul. Integr. Comp. Physiol..

[64] Tallis J., James R.S., Cox V.M., Duncan M.J. (2012). The Effect of Physiological Concentrations of Caffeine on the Power Output of Maximally and Submaximally Stimulated Mouse EDL (Fast) and Soleus (Slow) Muscle. J. Appl. Physiol..

[65] Fitts R.H., Widrick J.J. (1996). Muscle Mechanics: Adaptations with Exercise-Training. Exerc. Sport Sci. Rev..

[66] MacIntosh B.R., Herzog W., Suter E., Wiley J.P., Sokolosky J. (1993). Human Skeletal Muscle Fibre Types and Force: Velocity Properties. Eur. J. Appl. Physiol..

[67] Segerström A.B., Holmbäck A.M., Hansson O., Elgzyri T., Eriksson K.-F., Ringsberg K., Groop L., Wollmer P., Thorsson O. (2011). Relation between Cycling Exercise Capacity, Fiber-Type Composition, and Lower Extremity Muscle Strength and Muscle Endurance. J. Strength Cond. Res..

[68] Metaxas T.I., Mandroukas A., Vamvakoudis E., Kotoglou K., Ekblom B., Mandroukas K. (2014). Muscle Fiber Characteristics, Satellite Cells and Soccer Performance in Young Athletes. J. Sports Sci. Med..

[69] Paddon-Jones D., Leveritt M., Lonergan A., Abernethy P. (2001). Adaptation to Chronic Eccentric Exercise in Humans: The Influence of Contraction Velocity. Eur. J. Appl. Physiol..

[70] Shepstone T.N., Tang J.E., Dallaire S., Schuenke M.D., Staron R.S., Phillips S.M. (2005). Short-Term High- vs. Low-Velocity Isokinetic Lengthening Training Results in Greater Hypertrophy of the Elbow Flexors in Young Men. J. Appl. Physiol..

[71] Pickering C., Kiely J. (2018). Are the Current Guidelines on Caffeine Use in Sport Optimal for Everyone? Inter-Individual Variation in Caffeine Ergogenicity, and a Move Towards Personalised Sports Nutrition. Sports Med..

[72] Amaral G.M., Marinho H.V.R., Ocarino J.M., Silva P.L.P., de Souza T.R., Fonseca S.T. (2014). Muscular Performance Characterization in Athletes: A New Perspective on Isokinetic Variables. Braz. J. Phys. Ther..

[73] Burigo R.L., Scoz R.D., Alves B.M.d.O., da Silva R.A., Melo-Silva C.A., Vieira E.R., Hirata R.P., Amorim C.F. (2020). Concentric and Eccentric Isokinetic Hamstring Injury Risk among 582 Professional Elite Soccer Players: A 10-Years Retrospective Cohort Study. BMJ Open Sport Exerc. Med..

[74] Woolf K., Bidwell W.K., Carlson A.G. (2009). Effect of Caffeine as an Ergogenic Aid during Anaerobic Exercise Performance in Caffeine Naive Collegiate Football Players. J. Strength Cond. Res..

[75] Astorino T.A., Martin B.J., Schachtsiek L., Wong K., Ng K. (2011). Minimal Effect of Acute Caffeine Ingestion on Intense Resistance Training Performance. J. Strength Cond. Res. Natl. Strength Cond. Assoc..

[76] Kalmar J.M. (2005). The Influence of Caffeine on Voluntary Muscle Activation. Med. Sci. Sports Exerc..

[77] Meissner K. (2011). The Placebo Effect and the Autonomic Nervous System: Evidence for an Intimate Relationship. Philos. Trans. R. Soc. Lond. B. Biol. Sci..

[78] Hammadah M., Alkhoder A., Al Mheid I., Wilmot K., Isakadze N., Abdulhadi N., Chou D., Obideen M., O’Neal W.T., Sullivan S. (2017). Hemodynamic, Catecholamine, Vasomotor and Vascular Responses: Determinants of Myocardial Ischemia during Mental Stress. Int. J. Cardiol..

[79] Collin S., Sennoun N., Levy B. (2008). Cardiovascular and Metabolic Responses to Catecholamine and Sepsis Prognosis: A Ubiquitous Phenomenon?. Crit. Care Lond. Engl..

[80] Kubera B., Hubold C., Otte S., Lindenberg A.-S., Zeiss I., Krause R., Steinkamp M., Klement J., Entringer S., Pellerin L. (2012). Rise in Plasma Lactate Concentrations with Psychosocial Stress: A Possible Sign of Cerebral Energy Demand. Obes. Facts.

[81] Graham T.E., Helge J.W., MacLean D.A., Kiens B., Richter E.A. (2000). Caffeine Ingestion Does Not Alter Carbohydrate or Fat Metabolism in Human Skeletal Muscle during Exercise. J. Physiol..

[82] Mizuno M., Kimura Y., Tokizawa K., Ishii K., Oda K., Sasaki T., Nakamura Y., Muraoka I., Ishiwata K. (2005). Greater Adenosine A(2A) Receptor Densities in Cardiac and Skeletal Muscle in Endurance-Trained Men: A [11C]TMSX PET Study. Nucl. Med. Biol..

[83] Grgic J. (2021). Effects of Caffeine on Resistance Exercise: A Review of Recent Research. Sports Med..

[84] Santos Junior E.R.T., de Salles B.F., Dias I., Ribeiro A.S., Simão R., Willardson J.M. (2021). Classification and Determination Model of Resistance Training Status. Strength Cond. J..

